# The PEST (Pathology, Epidemiology, Severity, Treatment) approach to optimizing antimicrobial therapy

**DOI:** 10.1186/s12909-023-04286-1

**Published:** 2023-05-06

**Authors:** Kusha Davar, Tara Vijayan

**Affiliations:** Department of Infectious Diseases, David Geffen School of Medicine at UCLA, 10833 Le Conte Ave, Room 52-215 CHS, Los Angeles, CA 90095 USA

**Keywords:** Graduate medical education, Therapeutic reasoning, Antibiotic stewardship, Case-based learning, Infectious diseases

## Abstract

**Background:**

Selecting an empiric antimicrobial regimen can be difficult for early learners and misuse of antibiotics can lead to adverse events and antimicrobial resistance. There have been few interventions that have focused on improving antibiotic decision making, as a form of therapeutic reasoning, for post-graduate trainees. We describe here an approach to aid internal medicine interns in their therapeutic reasoning, particularly when it comes to diagnosing and empirically treating infections.

**Methods:**

The PEST (pathology, epidemiology, severity, treatment) model was created as a four-step approach to therapeutic reasoning and choosing an appropriate antimicrobial regimen for a given infectious disease syndrome. In February 2020, we conducted two independent teaching sessions for interns on the PEST approach. We assessed pre-and post-teaching responses to five clinical vignette-based questions. Results were presented as a percentage of interns who chose an appropriate antibiotic and provided sufficient therapeutic reasoning as defined by using at least three out of the four PEST criteria. Statistical analysis occurred via Fischer’s exact test to determine the level of statistical significance between responses.

**Results:**

Twenty-seven interns participated in the activity. At baseline, several interns had incorporated aspects of the PEST approach in their pre-teaching responses. Ten interns commented on the usefulness of such a systematic approach. While there was no statistically significant difference in antibiotic selection, the teaching session demonstrated a trend towards significance in improving therapeutic reasoning as defined by the PEST strategy.

**Conclusion:**

Our results suggested an improvement in using a structured cognitive tool such as the PEST approach to reinforce therapeutic reasoning, but the method did little to improve antibiotic selection. Some interns used select “PEST” concepts prior to the intervention suggesting that the PEST approach may enhance prior knowledge or clinical reasoning skills. Continued incorporation of the PEST approach using a case-based framework may solidify conceptual and practical knowledge of antimicrobial selection. Further studies are needed to assess the impact of such teaching interventions.

**Supplementary Information:**

The online version contains supplementary material available at 10.1186/s12909-023-04286-1.

## Practice points

As a goal, learners will be able to:

1. Understand the importance of therapeutic reasoning and selecting optimal empiric antibiotics
2. Apply a structured approach to selecting empiric antibiotics, Particularly at an early learner stage
3. Use therapeutic reasoning to arrive at the selection of empiric antibiotics

## Background

Selecting an empiric antimicrobial regimen for a given syndrome can be challenging for doctors-in-training who are by and large the main prescribing physicians in academic medical centers. Overuse of the so-called “broad spectrum antibiotics” is common and can result in adverse events, selection of resistance through disruption of the gut microbiota, and subsequent superinfection with opportunistic pathogens such as *Clostridioides difficile.*

Studies have shown that both residents and attending physicians in the general medicine and intensive care setting acknowledge the concern of antibiotic resistance and support the notion of antimicrobial stewardship programs [1]. Resources are often available in the hospital to guide clinicians to choose the correct antibiotics. However, such a task may be daunting in the acute setting, especially to less-experienced clinicians, residents, and medical students. Focusing on how to choose antibiotics, rather than what antibiotics to choose (i.e., what bug, what drug), allows for more nuanced therapeutic reasoning that can be applied across different scenarios [2].

Experts have described key decision-making points in antibiotic prescribing, but to date little has been written regarding specific methods to bridge gaps in teaching appropriate antibiotic prescribing across the spectrum of learners [3–6]. The Centers for Disease Control and Prevention (CDC) estimates that more than half of antibiotic prescribing for selected events in hospitals are not consistent with recommended prescribing practices [7]. As some of the most common prescribers in hospital systems, resident physicians should be considered for targeted antimicrobial stewardship educational interventions [6].

In order to allow for adaptability across clinical settings (rural versus urban, community versus university-based hospitals), it is important to teach the therapeutic reasoning behind empiric antibiotics [8–10] Several interventions previously have been studied to achieve improvement in improving diagnosis including electronic health record based clinical decision support tools and checklists [11, 12]. Given the particular nuance of therapeutic reasoning (which requires appropriate diagnosis and understanding of best available treatments), in this study we used an “instructions at test” cognitive reasoning tool, defined as interventions that instruct participants to use a certain reasoning strategy, where the instructions are provided together with the cases on which performance is measured, that is, the test cases [11].

Our intervention focuses on existing resources such as antibiograms, which can help educate early trainees in selecting appropriate antibiotics. We describe here a therapeutic reasoning strategy to aid doctors who are early in their training, particularly when it comes to empirically treating infections.

## Methods

### Overview of methods

**Fig. 1:**
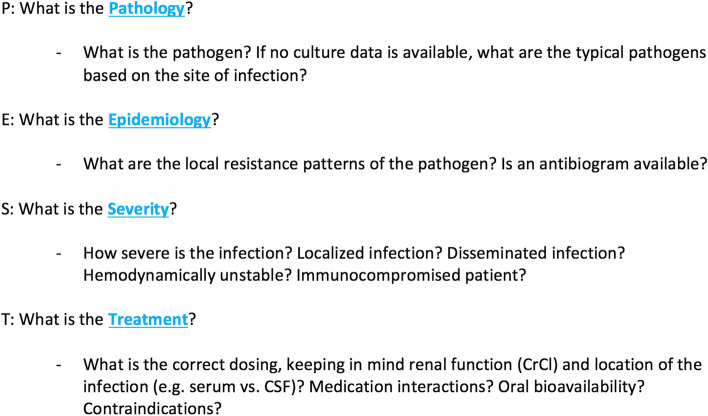
PEST approach

The PEST model was created as a four-step approach to therapeutic reasoning and choosing an appropriate antimicrobial regimen for a given infectious disease syndrome (Fig. 1). Parts of this method have been described in prior studies [9, 13]. PEST involves four steps that are integral to deciding on an antimicrobial regimen and serves as a checklist reminder for prescribers:

1. What is the pathology? (i.e., pathogen at hand or suspected pathogens)
2. What is the epidemiology? (i.e., local resistance patterns based on institutional antibiograms)
3. What is the severity of the disease? (i.e., both syndrome and host driven)
4. What is the treatment being chosen? (e.g., specific details surrounding the route of administration, dosing, medication interactions)

In February 2020, we conducted two independent teaching sessions for interns on the PEST approach during noon conference at UCLA Ronald Reagan Medical Center (UCLA RRMC) and the Greater Los Angeles Veterans Affairs Healthcare System (GLA VAHS). Each intern completed a pre-and post-intervention assessment which included free text responses to five clinical vignette-based questions (Additional file 1: Appendix 1). We developed a scoring rubric for these free text responses to assess both optimal selection of antibiotics and the use of therapeutic reasoning in medical decision making (Additional file 2: Appendix 2). Results were presented as the percentage of interns, both pre- and post-intervention, who chose an appropriate antibiotic and the percentage of interns who provided sufficient therapeutic reasoning as defined by using at least three out of the four PEST criteria. Additionally, we surveyed the interns on the perceived value of the intervention at the end of the session by asking them to offer comments.

### Development of assessment tool

Five questions were chosen to represent common infectious disease syndromes encountered in the hospital by internal medicine interns. The format of the questions was similar to board examination-style questions with a case-based vignette. However, the answer choices were free response, rather than multiple choice, to help simulate real-life clinical decision making. Each clinical vignette was designed to include conundrums encountered when prescribing antibiotics (e.g., acute renal failure, drug allergies, immunocompromised state), which also helped learners navigate through the PEST algorithm when deciding on the optimal antimicrobial regimen.

As is the case with real-life clinical decision making, more than one approach to prescribing antibiotics was deemed to be correct. Though not previously validated, the rubric was constructed based on a general knowledge of acceptable antibiotic choices for specific infectious disease syndromes and on an a priori construct of therapeutic reasoning [9, 13]. The rubric is simple and could be easily reproduced by other educators (Additional file 2: Appendix 2). Several antibiotic choices were acceptable for each clinical vignette question, determined by a consensus of infectious disease physicians. For therapeutic reasoning, the PEST approach was used as a rubric where points were awarded when interns described reasons for choosing antibiotics, defined by using at least three out of the four PEST criteria (e.g., description of pathology, epidemiology, severity, or treatment details).

### Development of teaching intervention

The teaching component and intervention during noon-conference consisted of 1.) an overview of the PEST approach and the rationale behind its use and 2.) a series of clinical vignette-style questions as a group with more in-depth discussions of how to approach each scenario with the PEST checklist kept in mind. The group was encouraged to use the institutional antibiogram to help make clinical decisions.

The session was one hour in duration with fifteen minutes for both the pre- and post-assessments and approximately thirty minutes allocated for teaching. The end of the hour consisted of one to two minutes dedicated for written feedback from the interns on the quality of the session. Upon conclusion, the interns repeated the assessment and submitted voluntary written evaluations. Each intern was assigned a number to anonymously group both pre- and post-intervention assessments and evaluations.

### Statistical methodology

A convenience sample was used based on availability at each respective site. A Fisher’s exact test was performed to calculate the statistical significance of the responses with a *p* value set to < / = 0.05. Responses were evaluated per question, and per hospital training site, to determine if the differences between interns’ responses were statistically significant pre- and post-teaching intervention.

## Results

**Fig. 2:**
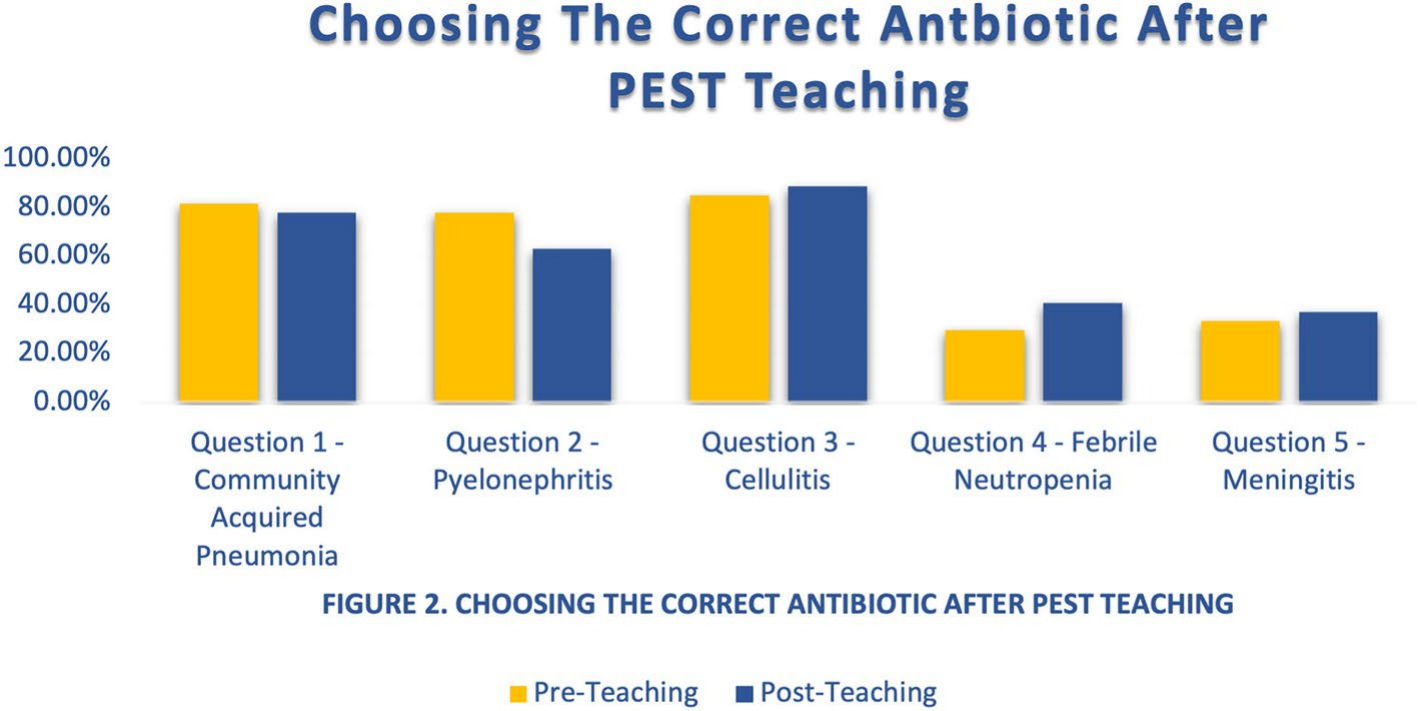
Differences in antibiotic choices

Twenty-seven interns participated in the activity, with sixteen interns present at the GLA VAHS session and eleven interns present at the UCLA RRMC session. When comparing correct antibiotic responses to each clinical vignette question, the difference in the number of interns at UCLA RRMC and GLA VAHS who answered appropriately pre- and post-intervention for questions 1 through 5 are described in Table 1. Notably, with question number 2 at the GLA VAHS and question numbers 1 and 2 at UCLA RRMC, more interns answered appropriately pre-intervention compared with post-intervention (Fig. 2). None of the interns answered all the antibiotic selection questions correctly (i.e., earned 100%) at either site both pre- and post-intervention. The differences in the pre and post intervention tests were not significant (Fig. 2).

**Table 1:** Differences in antibiotic choices after PEST teaching

	**UCLA RRMC**			**GLA VAHS**		
Questions	Pre	Post	*P*-value	Pre	Post	*P*-value
1 – Community Acquired Pneumonia	8/11 (72.7%)	6/11 (54.5%)	0.3297	14/16 (87.5%)	15/16 (93.8%)	0.5216
2—Pyelonephritis	8/11 (72.7%)	7/11 (63.6%)	0.5	13/16 (81.3%)	10/16 (62.5%)	0.2166
3—Cellulitis	10/11 (90.9%)	10/11 (90.9%)	0.8214	13/16 (81.3%)	14/16 (87.5%)	0.5023
4 – Febrile Neutropenia	4/11 (36.4%)	6/11 (54.5%)	0.335	4/16 (25%)	5/16 (31.3%)	0.5
5—Meningitis	5/11 (45.5%)	5/11 (45.5%)	0.665	4/16 (25%)	5/16 (31.3%)	0.5

**Fig. 3:**
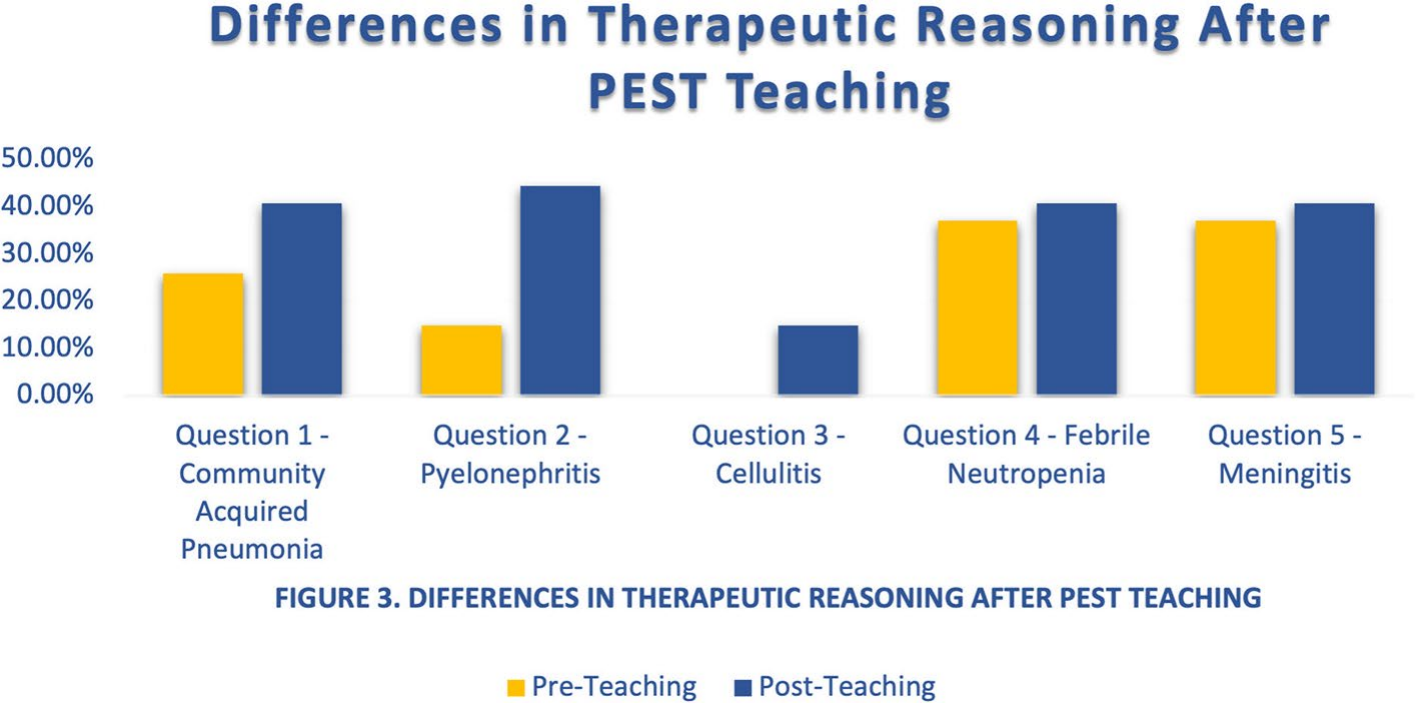
Differences in therapeutic reasoning

With respect to the therapeutic reasoning responses, the difference in the number of interns at UCLA RRMC and GLA VAHS who answered appropriately pre- and post-intervention for questions 1 through 5 are described in Table 2. None of the interns provided full therapeutic reasoning for each question at both sites both pre- and post-intervention. At baseline, ten out of sixteen interns at the at the GLA VAHS session and ten out of eleven interns at UCLA RRMC session had incorporated aspects of the PEST approach in their pre-intervention responses. Overall interns demonstrated an improvement in their therapeutic reasoning, but this was not statistically significant (Fig. 3).

**Table 2:** Differences in therapeutic reasoning after PEST teaching

	**UCLA RRMC**			**GLA VAHS**		
Questions	Pre	Post	*P*-value	Pre	Post	*P*-value
1 – Community Acquired Pneumonia	1/11 (9.1%)	5/11 (45.5%)	0.0743	6/16 (37.5%)	6/16 (37.5%)	0.642
2—Pyelonephritis	1/11 (9.1%)	5/11 (45.5%)	0.0743	3/16 (18.8%)	7/16 (43.8%)	0.1262
3—Cellulitis	0/11 (0%)	1/11 (9.1%)	0.5	0/16 (0%)	3/16 (18.8%)	0.1129
4 – Febrile Neutropenia	5/11 (45.5%)	5/11 (45.5%)	0.665	5/16 (31.3%)	6/16 (37.5%)	0.5
5—Meningitis	5/11 (45.5%)	4/11 (36.4%)	0.5	5/16 (31.3%)	7/16 (43.8%)	0.358

Interns were given the option to write in free-response format their impression of the session and the PEST approach. Ten interns commented on the usefulness of the approach. Specifically, interns appreciated the systematic approach to choosing antibiotics guided by reasoning, rather than reflexively choosing the correct antibiotic. Additionally, interns enjoyed reviewing the institution’s antibiogram both at the GLA VAHS and UCLA RRMC to help with decision making surrounding antibiotic choices. Lastly, one intern relayed that PEST provided a structured approach to what was informally taught in training, which can facilitate thoroughness when busy on clinical service.

## Discussion

Our results showed that the PEST approach did little to improve antibiotic selection, however the results suggest an improvement in therapeutic reasoning that was not statistically significant. The lack of improvement in antibiotic selection could be due to the timing of the intervention as this study was conducted in February of intern year. Many interns were very comfortable with common infectious disease syndromes such as community acquired pneumonia, pyelonephritis and cellulitis, but less comfortable with febrile neutropenia or meningitis which are in general rarer conditions on a general medicine service. Additionally, the lack of a significant improvement in therapeutic reasoning may have been due to the small sample size.

Although we initially designed the study with the goal of improving antibiotic selection, we soon realized that the true benefit may have been in improving therapeutic reasoning as a means to guide antibiotic selection across different clinical scenarios. Antibiotic selection for specific clinical syndromes may, in part, be informed by institutional and broader clinical guidance, which are perhaps easily “memorized” or accessed in different ways. At our institution, we offer some clinical decision support through a smartphone application known as *Firstline™* which includes antibiotic choices for select infectious disease syndromes in addition to details about our antibiogram [14].

Even though therapeutic reasoning may be an important intervention to help teach early learners how to choose antimicrobials wisely, meta-analyses have described that such focuses only provide a modest improvement in diagnostic accuracy [11, 12]. We hope that through continued implementation of structured cognitive tools such as PEST, therapeutic reasoning skills are integrated into trainees’ decision-making process. We believe that the PEST approach offers an example of a clinical reasoning strategy that learners may use when encountering the difficult task of choosing antibiotics.

In this study, we used the educational strategy of small group discussion and teaching in conjunction with clinical vignettes. We created an assessment to demonstrate mastery of the material for pre- and post-intervention. We graded the therapeutic reasoning responses using a specific rubric which may be biased towards a post intervention improvement. However, as mentioned, even when taken into account, aspects of PEST were inherent in pre-intervention clinical reasoning which helps to supports the ongoing teaching to reinforce knowledge that may already taught implicitly or explicitly in an existing medical curriculum [15]. Ultimately, continued incorporation of the PEST approach using a case-based framework may solidify conceptual and practical knowledge of antimicrobial selection.

Many clinicians do not have a specific approach to choosing antibiotics and often model behaviors based on prior experiences or training [5]. Our study assessed some of these gaps. For instance, fewer than 40% of interns correctly identified antibiotic choices for meningitis. Much of post-graduate education is acquired informally from senior members of the team, with formal didactics and conferences serving as a centralized resource for resident education. The PEST approach may serve to function as a targeted needs assessment to establish a baseline level of therapeutic reasoning for residents and may help to enrich such a skill through continuous implementation [16, 17].

To resemble real life decision making in infectious diseases, we chose to not create multiple choice questions, which are often administered as a method to ascertain clinical proficiency during standardized examinations. This allowed interns to explain their answer choices and learn from the explanations, rather than by simply choosing the correct response to a question. Antibiotic choices are not always obvious, and there may be several ways to treat a clinical syndrome. Open-ended questions may allow interns to acknowledge that clinical reasoning may sometimes be more important than arriving at a singular antibiotic choice.

Future studies should assess the value of interventions such as the PEST approach in a longitudinal curriculum at various points during the intern year, using a spiral curriculum approach. The spiral curriculum involves teaching a concept, in this case the PEST approach, in a longitudinal format with spaced repetition [18]. However, it is not simply the repetition of a topic, but in fact, a successive deepening of the subject upon each encounter to allow for reinforcement, integration, and the mastery of higher-level objectives [18]. This would allow for doctors in training to gain familiarity with therapeutic reasoning and recognize its benefits in choosing antimicrobial therapies.

We acknowledge several limitations with our study. For instance, the teaching sessions consisted only of a one-time intervention at each respective site. Additionally, the intervention occurred at a snapshot in time half-way through the academic year, where doctors-in-training may have a certain amount of confidence when compared to counterparts earlier, or later, in the academic year as mentioned above. Lastly, the cohort included doctors in training within internal medicine, which may not be generalizable for the entire population of recent graduates from medical school. More frequent teaching to a larger and diverse population, spread throughout the academic year, will be needed to reliably measure the efficacy of the PEST approach.

## Conclusions

In conclusion, the PEST approach may be effective in enriching therapeutic decision-making to guide antibiotic choices for doctors in training, though further research on this cognitive reasoning tool, with perhaps a longitudinal approach and a larger sample size, may be warranted to demonstrate a significant impact. By having such a framework, medical residents can have a reasoning strategy to selecting antibiotics for varied infectious disease syndromes across diverse clinical settings. We hope to continue to assess how to best utilize this intervention throughout the continuum of medical education in an effort to improve comfort with antibiotic prescribing.

## Supplementary Information

**Additional file 1:** **Appendix 1.** Clinical vignette questions.

**Additional file 2: Appendix 2.** Clinical vignette scoring rubric.

## Data Availability

All data generated or analyzed during this study are included in this published article [and its supplementary information files].

## Abbreviations

UCLA RRMC: UCLA Ronald Reagan Medical Center
GLA VAHS: Greater Los Angeles Veterans Affairs Healthcare System
