# Supplementary material for: The PEST (Pathology, Epidemiology, Severity, Treatment) approach to optimizing antimicrobial therapy
Source: BMC Med Educ. 2023 May 6;23:316. doi: 10.1186/s12909-023-04286-1 (PMC10163704; doi:10.1186/s12909-023-04286-1)
Supplement: Supplementary file 1 — Additional file 1: Appendix 1. Clinical vignette questions. [file 12909_2023_4286_MOESM1_ESM.docx]

The PEST Approach to Choosing Antimicrobial Medications

Clinical Vignette Questions

1. A 35-year-old man is evaluated for a 1-day history of fever, chills, vomiting, diarrhea, and cough productive of green and brownish sputum. He feels short of breath with minimal activity, and his breathing appears uncomfortable. He was diagnosed with influenza A two weeks prior which he reportedly improved one week ago. Medical history is significant for asthma for which he takes albuterol and IV drug use.

On physical examination, temperature is 39.0 °C, blood pressure is 100/60 mm Hg, pulse rate is 110/min, and respiration rate is 30/min. Oxygen saturation is 92% breathing ambient air. Pulmonary examination is notable for diffusely decreased breath sounds throughout and crackles at the right lung base; the remainder of the examination is unremarkable. White blood cell count is 17,000/mm^3^ and creatinine is 3.5 mg/dL (baseline 0.9 mg/dL). Blood cultures taken peripherally are growing GPCs in clusters. Chest radiograph shows a right lower lobe infiltrate suggestive of consolidation pneumonia.

What antibiotic regimen would you choose? Please explain your answer in detail.

1. A 92-year-old woman is evaluated in the emergency department for fevers, chills, nausea, back pain, and dysuria. Symptom onset began 3 days prior with increased urinary frequency. She has a past medical history of atrial fibrillation and is currently taking warfarin.

On physical examination, she appears uncomfortable. Temperature is 38.6 °C, blood pressure is 110/60 mm Hg, and pulse rate is 120/min. Abdominal examination demonstrates diffuse tenderness, prominently in the suprapubic region with marked CVA tenderness. White blood cell count is 14,000/mm^3^ and renal function is within normal range. Blood cultures are drawn.

What antibiotic regimen would you choose? Please explain your answer in detail.

1. A 45-year-old man is seen by his primary care physician in clinic with an erythematous and painful right lower extremity. He reports having a spider bite on his right calf that he scratched and noticed a worsening rash, that is now well-demarcated and raised. He reports low grade fevers and chills. His past medical history is significant for poorly controlled type-2-diabetes and hypertension. He takes lisinopril 40 mg daily and 25 units of insulin glargine nightly.

On examination, he is afebrile with a blood pressure of 130/90 mm Hg and pulse rate of 80/min. His extremity exam is significant for an erythematous right lower extremity that is tender to palpation. Additionally, there is a 3 cm tender induration on his calf that is fluctuant on exam, suggestive of an underlying abscess. In the emergency department, his MRSA nares PCR is positive and white blood cell count is 15,000/mm^3^.

What antibiotic regimen would you choose? Please explain your answer in detail.

1. A 67-year-old woman with AML having recently undergone chemotherapy presents with fevers and chills. She has had loose stools over the past several days, reporting 3-4 watery, non-bloody bowel movements per day and cramping abdominal pain. She has recently completed a seven-day course of clindamycin for aspiration pneumonia.

On examination, she is febrile to 39.2 °C with a blood pressure of 130/90 mm Hg and pulse rate of 90/min. She is ill appearing. Pulmonary examination demonstrates clear lungs, however abdominal examination is significant for diffuse abdominal tenderness, without rebound or guarding. White blood cell count is 1,000/mm^3^ with an absolute neutrophil count of 400/mm^3^. Blood cultures have been drawn with a plan to start empiric therapy.

What antibiotic regimen would you choose? Please explain your answer in detail.

1. A 22-year-old man is brought in by ambulance and evaluated in the emergency department for altered mental status. He was found in his dorm room obtunded and warm to the touch. He has no known past medical history, however, is allergic to penicillin, which he has previously reported has caused a rash 3 days after starting an antibiotic for a skin infection.

On examination, he is A&O x 0, making incoherent noises. Temperature is 40.0 °C with a blood pressure of 110/70 mm Hg and pulse rate of 125/min. There is nuchal rigidity with photophobia. Cardiac examination reveals tachycardia and pulmonary examination is unremarkable. There are no rashes present. White blood cell count is 18,000/mm^3^ and platelets are 75,000/mm^3^. The remainder of the complete blood count and chemistry panel is unremarkable.

What antibiotic regimen would you choose? Please explain your answer in detail.
