# Supplementary material for: The PEST (Pathology, Epidemiology, Severity, Treatment) approach to optimizing antimicrobial therapy
Source: BMC Med Educ. 2023 May 6;23:316. doi: 10.1186/s12909-023-04286-1 (PMC10163704; doi:10.1186/s12909-023-04286-1)
Supplement: Supplementary file 2 — Additional file 2: Appendix 2. Clinical vignette scoring rubric. [file 12909_2023_4286_MOESM2_ESM.docx]

The PEST Approach to Choosing Antimicrobial Medications

Clinical Vignette Scoring Rubric

Two Different Grading Rubrics:

1. Did it change antibiotic choice?
2. Was the correct antibiotic chosen?
3. Did it change therapeutic reasoning?
4. Was PEST used to provide therapeutic reasoning?

Antibiotic Choice:

Question 1

1. Vancomycin/Linezolid – 1 point
2. Daptomycin, TMP/SMX, Doxycycline, or Clindamycin monotherapy – 0 points
3. Use of Ceftriaxone or CAP coverage OK (no points awarded or lost)

Question 2

1. Ceftriaxone, Ertapenem, Cefepime – 1 point
2. Pip/tazo, Meropenem, or Fluoroquinolone – 0 points

Question 3

1. Vancomycin, Cefazolin, Cephalexin, TMP/SMX, Doxycycline, Linezolid, Clindamycin – 1 point
2. Pip/tazo, Ertapenem, Meropenem – 0 points

Question 4

1. Pip/tazo, Cefepime, Meropenem AND oral vancomycin – 1 point
2. Absence of BOTH combinations above – 0 points
3. Vancomycin IV OK if mentioned for initial empiric but removed after 48 hours, if not – 0 points

Question 5

1. Vancomycin + Ceftriaxone – 1 point
2. Absence of BOTH combinations above – 0 points
3. Pip/tazo, Ertapenem, Meropenem – 0 points
4. Acyclovir IV OK (no points awarded or lost)

PEST Use (Requires 3 out of 4 Components of PEST to earn 1 point):

Question 1

1. P: MSSA/MRSA
2. E: Antibiogram Use
3. S: Bacteremia/Septic
4. T: AKI Acknowledgement or No Daptomycin Use Acknowledgement

Question 2

1. P: GNR (Urinary Pathogens)
2. E: Antibiogram Use
3. S: Pyelonephritis/Sepsis
4. T: Med-Med Interactions with warfarin or Inappropriateness of nitrofurantoin

Question 3

1. P: MSSA/MRSA, Streptococcus
2. E: Antibiogram Use
3. S: Cellulitis (Not Necessarily Sick)
4. T: AKI or K+ risk with Lisinopril and DM

Question 4

1. P: GNR (Pseudomonas)
2. E: Antibiogram Use
3. S: Neutropenic Fever/Sick
4. T: Pseudomonal Coverage + Oral C. Diff Coverage

Question 5

1. P: Strep. pneumoniae, Neisseria meningitidis, Hemophilus influenzae
2. E: Antibiogram Use
3. S: Meningitis/Sick
4. T: Address B-lactam allergy or Address CNS dosing (BBB penetration)
